# Community Characteristics and Population Dynamics of an Endangered *Rhododendron nymphaeoides*

**DOI:** 10.1002/ece3.71268

**Published:** 2025-04-10

**Authors:** Jun Luo, Xiaoyong Dai, Jin Chen, Congjun Yuan, Haodong Wang, Dali Luo

**Affiliations:** ^1^ Guizhou Academy of Forestry Guiyang Guizhou China; ^2^ College of Forestry Guizhou University Guiyang Guizhou China; ^3^ Key Laboratory of National Forestry and Grassland Administration on Biodiversity Conservation in Karst Mountainous Areas of Southwestern China Guiyang Guizhou China; ^4^ Guizhou Libo Karst Forest Ecosystem National Observation and Research Station Libo Guizhou China

**Keywords:** community structure, endangered, population dynamics, *Rhododendron nymphaeoides*, survival curve

## Abstract

*Rhododendron nymphaeoides* belongs to the Fortunea subsection of the Rhododendron genus. Despite being officially listed as an endangered species (with the conservation status of EN) by the International Union for Conservation of Nature Red List, The Red List of Rhododendrons, Red List of China's Higher Plants, and the Threatened Species List of China's Higher Plants, little is known about this species' community characteristics and population dynamics. This lack of knowledge hampers the conservation and management of its wild resources. This study was conducted on all existing distribution points of *R. nymphaeoides* through field investigations, revealing the species composition and community structure of its populations. This included analyzing the diameter class structure, creating life tables and survival curves, and analyzing population dynamics indices to shed light on the dynamics of the *R. nymphaeoides* populations. The results showed: (1) the four communities collectively comprise 122 species of vascular plants belonging to 55 families and 79 genera, with tropical elements predominantly influencing the community structure and environmental formation. Community IV exhibited the highest species diversity, Community III had the most even distribution of species, and Community II had the lowest species diversity. (2) A total of 288 *R. nymphaeoides* individuals were recorded, showing a spatially clustered distribution. (3) The age structure of the HSC population exhibits a reverse J‐shape, indicating a growing population, but there are still constraints in the transition from seedlings to saplings; the LSK population has a substantial number of young individuals and the highest proportion of middle‐aged individuals, characterizing it as a stable population; whereas the LZH and HTS populations have few young individuals and a high proportion of middle‐aged individuals, with an age structure that is spindle‐shaped and trending toward aging, placing these populations at significant risk of decline. (4) The survival curves for all four populations were of the Deevey‐II type, characterized by high mortality rates among seedlings. (5) The primary reasons for the difficulty in population renewal and development are the extremely low natural germination rates of seeds, weak competitiveness of seedlings, growth restricted to limestone mountain areas above 1500 m with thin soil layers, and frequent human disturbances. Therefore, conservation strategies that integrate in situ conservation, ex situ conservation, and reintroduction are recommended. Emphasis should be placed on long‐term monitoring of *R. nymphaeoides* populations and communities, enhanced management during the sapling stage, and efforts in seedling propagation and population restoration to achieve population strengthening and the natural reintroduction of artificially cultivated populations, thereby maintaining the population in a healthy state.

## Graphical abstract

*Rhododendron nymphaeoides* is explicitly listed as an endangered species in the “the International Union for Conservation of Nature's Red List,” “The Red List of Rhododendrons,” “Red List of China's Higher Plants,” and “Threatened Species List of China's Higher Plants.” It is also listed as a provincial‐level key protected wild plant in Sichuan, with few individuals in the wild and significant conservation value. This study conducted field surveys at all existing distribution sites of *R. nymphaeoides*, revealing the species composition and characteristics of the communities. By analyzing the diameter class structure of *R. nymphaeoides* populations, constructing static life tables and survival curves, and examining population dynamic change indices, the dynamics of *R. nymphaeoides* populations were revealed, suggesting a declining trend. The evaluation results show that *R. nymphaeoides* has not been well protected. There should be an emphasis on in situ conservation, particularly the management of the sapling stage, and attention should be given to long‐term monitoring of *R. nymphaeoides* populations and communities. Additionally, efforts should be made in seedling propagation and population restoration to maintain the populations in good condition.

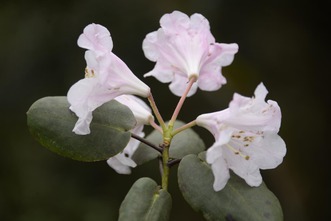

## 1 Introduction

In the 20th century, the rate of biodiversity loss caused by humans was 30 to 120 times faster than the average extinction rate recorded in the Cenozoic fossil record, earning it the label of the “Sixth Mass Extinction” (Pereira et al. 2024). Shocking statistics reveal that approximately 30% of tree species worldwide are on the brink of extinction, with at least 142 species already extinct in the wild. It is estimated that in the coming decades, biodiversity will be lost at a rate of 0.92%–5.1% per decade (Sun et al. 2023). Clearly, endangered species, as priority groups for biodiversity conservation, are the focal point of biodiversity protection (Ansari 2023).

**FIGURE 1:**
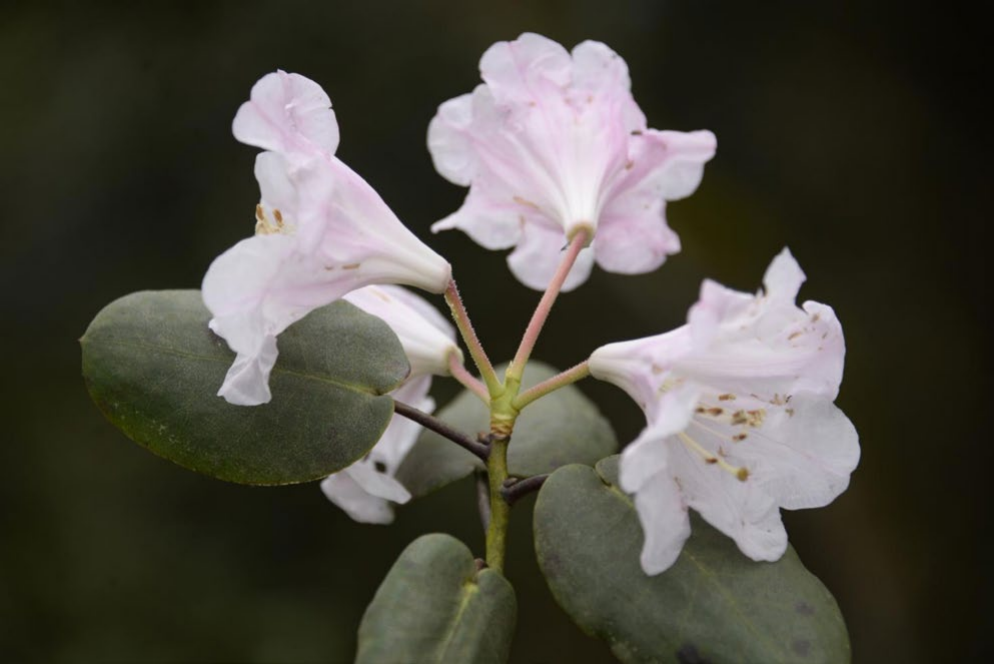
A photograph of *R. nymphaeoides*.

*Rhododendron nymphaeoides* W. K. Hu, belongs to the Fortunea subsection of the Rhododendron genus, with oval‐shaped leaves that have ear‐shaped bases, and its ovary and style covered in reddish‐brown glandular hairs, setting it apart from other species (Figure 1) (Dai et al. 2019, 2022). According to the “Red List of Threatened Species” published by the International Union for Conservation of Nature, the “Red List of Rhododendrons” co‐issued by the International Plant Protection Alliance, World Wildlife Fund, Global Trees Campaign, and Royal Botanic Garden Edinburgh, as well as the “Red List of China's Higher Plants” published by the Ministry of Ecology and Environment of China, and the “Threatened Species List of China's Higher Plants” published by the Chinese Academy of Sciences, *R. nymphaeoides* has been classified as Endangered with the criteria B1ab (i–iii). It is limited to the type locality in southern Sichuan (Gulin) and is surviving with only about a half dozen plants in a highly disturbed habitat on the highest peak in Guizhou, Wumeng Shan, representing one of the 19 globally endangered Rhododendron species (Ibbs et al. 2011; Liu et al. 2020). Over nearly a decade of survey and research, four wild populations dominated by *R. nymphaeoides* have been discovered. These include the Longzhaohe (LZH) and Hutoushan (HTS) populations in Gulin County, Sichuan Province; the Luosike (LSK) population in Duyun City, Guizhou Province; and the Heshancun (HSC) population in Dafang County, Guizhou Province. This species is only found at elevations above 1500 m on limestone mountains, covering a total area of approximately 2 ha with about 300 individual plants, indicating a very small population size. It is listed as a provincially protected plant in Sichuan and urgently requires corresponding conservation efforts (Dai et al. 2022).

The composition and structure of community species are crucial for classifying community types, measuring community stability, and predicting community dynamics. These attributes reflect the type of community, successional stage, stability, and environmental characteristics (including natural factors and human disturbances) to some degree. Population structure and dynamics form the core of population ecology research, playing a vital role in elucidating the survival status and regeneration strategies of endangered species, as well as in the maintenance of ecosystems (Wang et al. 2024). Therefore, the characteristics of populations and communities are the most critical basis for assessing the situation of endangered species and predicting their dynamic changes. These aspects are significantly important for understanding the causes of endangerment and optimizing conservation measures (Zhan et al. 2023). In recent years, scholars from around the world have conducted research on the community structure and population dynamics of endangered plants such as the *Acer miaotaiense* (Wang et al. 2024), *Taxus cuspidata* (Chang et al. 2024), *Camellia granthamiana* (Lin et al. 2023), *Rhododendron griersonianum* (Zhu et al. 2024), *Rhododendron aureum* (Jin et al. 2017), and *Rhododendron changii* (Yang et al. 2019). Based on these studies, conservation and management strategies have been proposed.

Currently, there is limited research on *R. nymphaeoides*, and studies focused on its community characteristics and population dynamics are even rarer. This research is fundamental for species protection and population restoration. In this context, the study investigates the community structure, diameter class structure, life table, and population survival curves of the species, based on a detailed survey of all existing populations and communities. The study also forecasts population changes over time. The objectives are to explore: (1) Whether the natural populations of *R. nymphaeoides* face a “bottleneck” phase in their renewal and maintenance? (2) Whether the populations of *R. nymphaeoides* show signs of decline under current habitat conditions? The results will reveal the current survival status of the species' communities, the structural characteristics of its populations, and dynamic changes in its numbers, thus providing a scientific basis for the rational and effective conservation of *R. nymphaeoides*.

## 2 Materials and Methods

### 2.1 Overview of the Study Area

**FIGURE 2:**
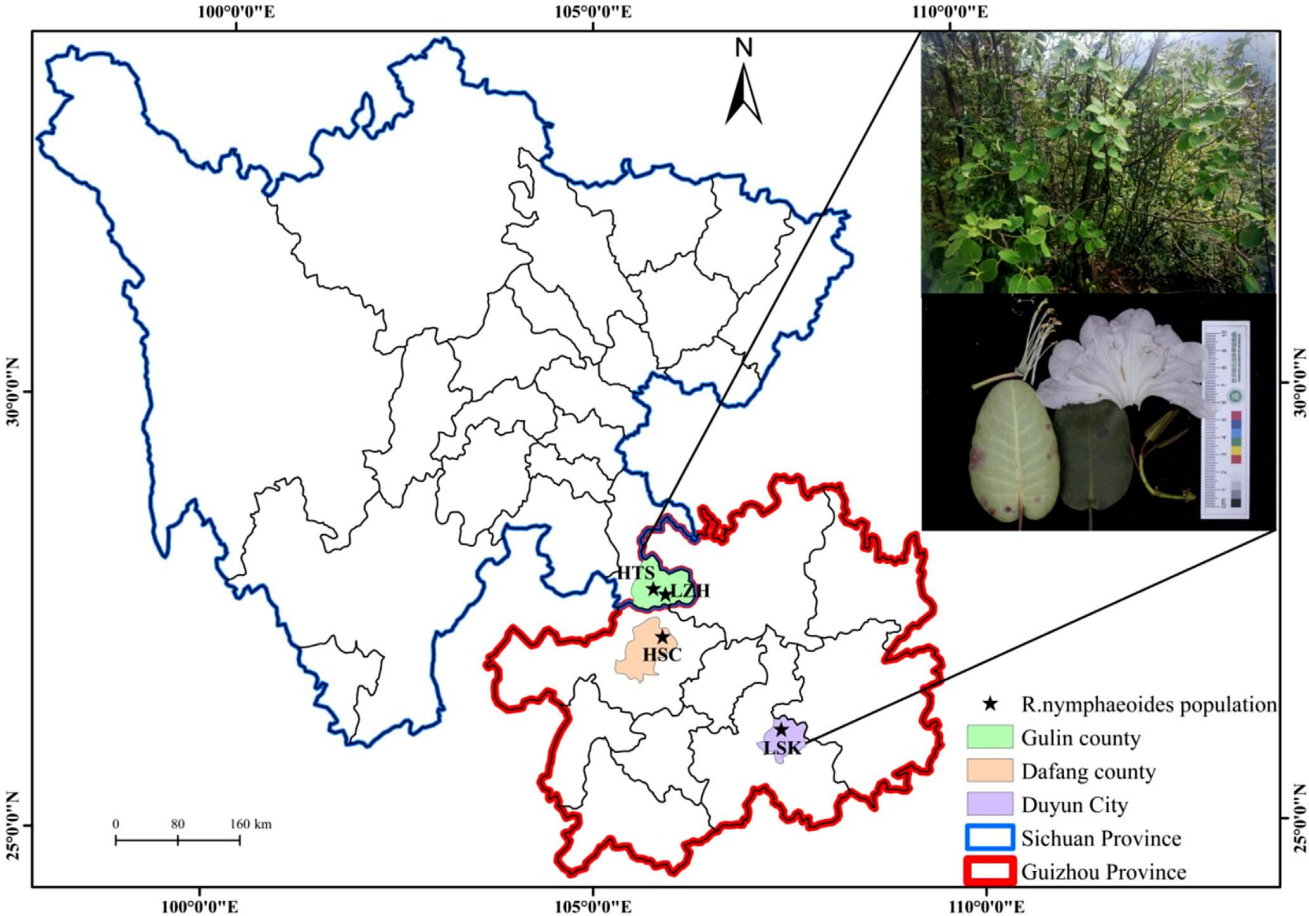
Geographic locations of *R. nymphaeoides* populations sampled in this study.

This survey covers all the natural communities of *R. nymphaeoides*, including those in Gulin County, Sichuan Province, Dafang County, Guizhou Province, and Duyun City (Figure 2). Gulin County is located in the transition zone between the Sichuan Basin and the Yunnan‐Guizhou Plateau, on the northern side of the Wumeng Mountain Range. It is situated in the lower reaches of the Chishui River Basin and belongs to a typical hilly and low‐to‐middle mountain terrain zone around the basin. The climate is strongly three‐dimensional, with distinct seasons, simultaneous rain and heat, an average annual temperature of 13.0°C–18.6°C, annual precipitation of 700–800 mm, and a frost‐free period of about 260 days. The area has moderate humidity, ample sunlight, and a subtropical monsoon climate. The soil is mainly purple soil (Xiao 2010). Dafang County lies in the transitional slope zone between the northwestern Guizhou Plateau (Yunnan‐Guizhou Plateau) and the hilly region of central Guizhou. It is located at the junction of the Chishui River Basin and the Wujiang River Basin and belongs to a middle mountain terrain type. The climate is mild with abundant rainfall, an average annual temperature of 11.8°C, and annual precipitation of 1155 mm. It is classified as a subtropical humid monsoon climate. The soil is primarily acidic lime soil, and the area is a typical karst landscape (Zhang 2022). Duyun City is located on the southeastern slope of the Yunnan‐Guizhou Plateau, at the junction of the Hongshui River Basin and the Yuanjiang River Basin, and belongs to a low mountain terrain type. The climate is mild and humid, with plentiful rainfall, an average annual temperature of 16.1°C, and annual precipitation of 1431 mm. It also has a subtropical humid monsoon climate. The soil is mainly yellow soil and lime soil, and the area is a karst landscape with many underground rivers and caves (Zhou 2020).

### 2.2 Community and Population Survey

Building upon the preliminary surveys, we identified four concentrated communities of the *R. nymphaeoides*, including the Longzhaohe population (LZH) and Hutoushan population (HTS) in Gulin County, Sichuan Province; the Luosike population (LSK) in Duyun City, Guizhou Province; and the Heshancun population (HSC) in Dafang County, Guizhou Province. Consequently, we conducted surveys in these four communities in September 2024. Drawing on the community ecological survey methods used for endangered species such as *R. griersonianum* (Zhu et al. 2024), *R. aureum* (Jin et al. 2017), and *R. changii* (Yang et al. 2019), a 400 m^2^ plot is considered sufficient to reflect the main characteristics of the population and the community it inhabits (Zhang et al. 2022a). We established 400 m^2^ plots (20 m × 20 m) at each of the four distribution points of *R. nymphaeoides* (Table 1). Each plot was subdivided into four 10 m × 10 m tree quadrats where every tree inside the quadrat was inspected. We recorded detailed information on each tree, including species name, diameter at breast height (DBH), tree height, and crown diameter. In the center of each tree quadrat, a 5 m × 5 m shrub quadrat was set up, where we recorded the species name, number of bushes (including saplings), height, and crown diameter of each shrub. In the center of the shrub quadrat, a 1 m × 1 m herbaceous plot was established where we documented the species name, number, height, and cover of each herb (including ferns). Additionally, using population ecology survey methods, we surveyed all *R. nymphaeoides* individuals within these four plots, recording their basal diameter (BD), height, and location information to further analyze the population structure and dynamics of *R. nymphaeoides* (Zhang et al. 2022b). Moreover, we collected specimens of *R. nymphaeoides* from the four plots and identified them through consultations with “Flora of China” and specimen comparisons at the Herbarium of the Kunming Institute of Botany, Chinese Academy of Sciences. The formal identification of the samples was performed by the plant taxonomist, Xiao‐Yong Dai. Voucher specimens have been deposited in the Herbarium of the Guizhou Provincial Academy of Forestry (GF), with deposition numbers DY20210411, DF20220428, GL20240922‐1, and GL20240922‐2. Our field investigations and experimental studies complied with the regulations of local legislative bodies and national and international guidelines.

**TABLE 1:** The sampling information of four populations of *R. nymphaeoides*.

No.	Community	Population	Locality	Longitude (E)	Latitude (N)	Altitude (m)	Gradient (°)	Aspect	Slope position	Canopy density (%)	Soil type	Soil thickness (cm)	Disturbance intensity
1	I	LSK	DuYun, GuiZhou	107°23′	26°15′	1642.88	35	Southeast	Uphill	65	Lime soil	20	Moderate
2	II	HSC	DaFang, GuiZhou	105°53′	27°25′	1696.42	50	Northwest	Uphill	80	Lime soil	20	Mild
3	III	LZH	GuLin, SiChuan	105°48′	28°06′	1559.21	40	Northwest	Uphill	75	Purple soil	25	Moderate
4	IV	HTS	GuLin, SiChuan	105°45′	28°07′	1707.58	47	Northwest	Ridge	80	Purple soil	25	Moderate

### 2.3 Calculation of Community Diversity Index and Importance Value

The main species composition of the community was analyzed by calculating the importance value. Five indices were selected to analyze community diversity: Margalef's richness index, Simpson dominance index, Shannon‐Wiener diversity index, Pielou evenness index, and Jaccard similarity index (Hao et al. 2021). The details are as follows:

(1) $${\text{Margalef}}^{'}s\;\text{richness}\;\text{index}:R=S-1/\ln N$$

(2) $$\text{Simpson}\;\text{dominance}\;\text{index}:D=1-\sum_{i=1}^{s}{P_{i}}^{2}$$

(3) $$\text{Shannon}‐\text{Wiener}\;\text{diversity}\;\text{index}:H^{\prime }=-\sum_{i=1}^{s}P_{i}\ln P_{i}$$

(4) $$\text{Pielou}\;\text{evenness}\;\text{index}:J_{e}=H^{\prime }/\ln S$$

(5) $$\text{Jaccard}\;\text{similarity}\;\text{index}:\mathrm{Sj}=\frac{e}{a+b+c+d-e}$$

(6) $$\text{Importance Value}=\left(\text{Relative}\;\text{Density}+\text{Relative}\;\text{Frequency}+\text{Relative}\;\text{Dominance}\right))/3$$

*S* is the total number of species in the plot, *N* is the total number of individuals in the plot, *P*_*i*_ is the proportion of individuals of the *i*th species in the plot, *a* is the number of species in community I, *b* is the number of species in community II, *c* is the number of species in community III, *d* is the number of species in community IV, and *e* is the number of species common to all four communities.

### 2.4 Population Age Structure Classification

Numerous studies have shown that, within the same environmental conditions, diameter classes and age classes often exhibit a positive correlation for the same species of plants. For rare and endangered woody plants, the “space‐for‐time” substitution method is commonly used; this involves using diameter class to approximate age class to delineate age structure (Li et al. 2021, 2022; Lin et al. 2023; Frost and Rydin 2000). Due to the multiple branching patterns of the trunk of *R. nymphaeoides*, BD is used as a criterion for age classification (Liu et al. 2015). Based on the recorded distribution of BDs of *R. nymphaeoides* and combined with field observations of its growth and development, the BD is divided into seven classes: BD1 ≤ 0.5 cm, 0.5 cm < BD2 ≤ 2 cm, 2 cm < BD3 ≤ 4.5 cm, 4.5 cm < BD4 ≤ 9.5 cm, 9.5 cm < BD5 ≤ 14.5 cm, 14.5 cm < BD6 ≤ 19.5 cm, and BD7 > 19.5 cm. Here, BD1 corresponds to age class I, BD2 corresponds to age class II, and so forth. Following these classification standards, the number of individuals in each age class of *R. nymphaeoides* is counted in order to conduct analyses of population structure characteristics and dynamics, and to construct life tables based on static data.

### 2.5 Population Spatial Structure

There are three types of population spatial structure: random distribution, uniform distribution, and clustered distribution. The degree of aggregation in the spatial distribution pattern of the *R. nymphaeoides* population was analyzed using methods such as the variance‐to‐mean ratio, negative binomial index (*K*), diffusion index (*C*), clumping index (*I*), mean crowding (M*), and patchiness index (*P*) (Pan et al. 2018; Xu et al. 2016). The formula is as follows:

$X=\sum N_{i}=1X_{i}/N$, $S^{2}=\sum N_{i}=1\left(\mathrm{Xi}-X\right)/N-1$, in the formula, *N* is the number of basic subplots, and *X*_*i*_ is the number of individuals in the *i*th subplot.

*K* = $X^{2}/\left(S^{2}-X\right)$: When *K* < 0, the population exhibits a uniform distribution; when *K* > 0, it exhibits a clumped distribution; when *K* > 8, it exhibits a random distribution.

*C* = $S^{2}/X$: When *C* = 1, the population exhibits a random distribution; when *C* > 1, it exhibits a clumped distribution; when *C* < 1, it exhibits a uniform distribution.

*I* = $S^{2}/X-1$: When *I* = 0, the population exhibits a random distribution; when *I* > 0, it exhibits a clumped distribution; when *I* < 0, it exhibits a uniform distribution.

M* = $X+S^{2}/X+1$: When M* > *X*, the population exhibits a clumped distribution; when M* = *X*, the population exhibits a random distribution; when M* < *X*, the population exhibits a uniform distribution.

*P* = $M^{*}/X$: When *P* = 1, the population exhibits a random distribution; when *P* > 1, it exhibits a clumped distribution; when *P* < 1, it exhibits a uniform distribution.

### 2.6 Static Life Tables

The static life table can reflect the mortality and survival processes of a population, providing foundational data for analyzing population dynamics. Based on the aforementioned diameter class classification standards, a static life table was constructed (Jiang 1992). The relevant parameters are as follows:

(7) $$l_{x}=\frac{a_{x}}{a_{0}}\times 1000$$

(8) $$d_{x}=l_{x}-l_{x+1}$$

(9) $$q_{x}=\frac{d_{x}}{l_{x}}\times 100\%$$

(10) $$L_{x}=\left(l_{x}+l_{x+1}\right)/2$$

(11) $$T_{x}=\sum_{x=1}^{n}L_{x}$$

(12) $$e_{x}=\frac{T_{x}}{l_{x}}$$

(13) $$S_{x}=\frac{l_{x+1}}{l_{x}}$$

In the formula, *x* represents the age class, *a*_*x*_ is the number of individuals in age class *x* after smoothing, and *a*_0_ is the number of individuals in age class I after smoothing; *l*_*x*_ is the number of surviving individuals at the beginning of age class *x*, standardized (usually converted to 1000); *d*_*x*_ is the standardized number of deaths between age class *x* and *x* + 1; *q*_*x*_ is the mortality rate between age classes *x* and *x* + 1; *L*_*x*_ is the number of individuals still alive during the interval between age classes *x* and *x* + 1, or the interval life expectancy; *T*_*x*_ is the total number of individuals from age class *x* onward; *e*_*x*_ is the expected lifespan or mean life expectancy of individuals entering age class *x*; *S*_*x*_ is the survival rate; and *K*_*x*_ is the population extinction rate.

### 2.7 Survival Curve Fitting

The survival curve is a graph that reflects the survival status of individuals in each age class of a population, typically divided into three types (Long et al. 2021). Type I is a convex curve, indicating that few individuals die before reaching their physiological lifespan; Type II is diagonal, indicating equal mortality at all ages; and Type III is concave, indicating a higher number of early deaths (Deevey 1947). In this study, we used exponential equations ($N_{x}=N_{0}e^{-\mathrm{bx}}$) and power function equations ($N_{x}=N_{0}x^{-b}$) to test the types of survival curves (Hett and Loucks 1976), where *N*_*x*_ is the number of surviving individuals of age class *x* after smoothing, *N*_0_ is the initial number of individuals in the population, and *b* is the mortality rate. The fit was assessed using the coefficient of determination (*R* ^2^) and *F*‐test values.

### 2.8 Population Viability Analysis

To further reveal the structural and dynamic changes of *R. nymphaeoides* population, population survival rate (*S*_*i*_), cumulative mortality rate (*F*_*i*_), mortality density (*f*_*ti*_), and hazard rate (*λ*_*ti*_) were introduced for survival analysis of the population (Yuan et al. 2022), as shown in the following formulas:

(14) $$S_{i}=S_{1}\times S_{2}\times S_{3}\cdots \times S_{x}$$

(15) $$F_{i}=1-S_{i}$$

(16) $$f_{\mathrm{ti}}=\frac{S_{i-1}-S_{i}}{h_{i}}$$

(17) $$\lambda_{\mathrm{ti}}=\frac{2\left(1-S_{i}\right)}{h_{i}\left(1+S_{i}\right)}$$

In the formula, *h*_*i*_ represents the age class width.

### 2.9 Population Dynamics Quantification

The age‐class dynamics of plant populations reflect the population's response to external disturbances. This is commonly applied in the study of endangered plant population structure and dynamics, providing valuable guidance for population management under external disturbances. A quantitative analysis method was used to analyze the population dynamics of this species (Liu et al. 2023), with the following specific calculation formula:

① Age‐class dynamic index between populations.

(18) $$V_{n}=\frac{S_{n}-S_{n+1}}{\max\left(S_{n},S_{n+1}\right)}$$

② Population structure dynamic index when external disturbances are ignored.

(19) $$V_{\mathrm{pi}}=\frac{1}{\sum_{n=1}^{k-1}S_{n}}\cdot \sum_{n=1}^{k-1}\left(S_{n}\cdot V_{n}\right)$$

③ Population structure dynamic index when external disturbances are considered.

(20) $$V^{\prime }\mathrm{pi}=\frac{\sum_{n=1}^{k-1}\left(S_{n}\cdot V_{n}\right)}{K\cdot \min\left(S_{1},S_{2},S_{3}\cdots S_{k}\right)\cdot \sum_{n=1}^{k-1}S_{n}}$$

④ Risk probability that the population bears from external disturbances.

(21) $$P_{\max}=\frac{1}{K\cdot \min\left(S_{1},S_{2},S_{3}\cdots S_{k}\right)}$$

In the formulas above (①, ②, ③, ④), *V*_*n*_ represents the dynamic index of individual number changes from age class *n* to *n* + 1; *S*_*n*_ and *S*_*n*_ + 1 represent the number of individuals in age class *n* and age class *n* + 1, respectively; *K*/*k* represents the population size magnitude; and the maximum and minimum values are taken from *S*_*n*_, *S*_*n*+1_, and *S*_*k*_ within the parentheses. *V*_*pi*_ is the quantity change dynamic index of the entire population structure without considering external disturbances; $V^{\prime }\mathrm{pi}$ is the quantity change dynamic index of the entire population structure when external disturbances are considered. When *V*_*n*_, *V*_*pi*_, and $V^{\prime }\mathrm{pi}$ are the positive values, it indicates that the population size is growing. When *V*_*n*_, *V*_*pi*_, and $V^{\prime }\mathrm{pi}$ are zero, it indicates that the population size is in a stable state. When *V*_*n*_, *V*_*pi*_, and $V^{\prime }\mathrm{pi}$ are the negative values, it indicates that the population size is declining.

### 2.10 Time Series Forecasting

This study uses the moving average method to predict the age structure of the population of *R. nymphaeoides* in four sample sites (Jiang 1992). The specific calculation formula is as follows:

(22) $$M_{t}^{\left[1\right]}=\frac{1}{n}\sum_{k=t-n+1}^{t}X_{k}$$

In the formula, *n* represents the time to be predicted; *t* is the age class; $M_{t}^{\left[1\right]}$ is the population size of age class t in *n* years; and *X*_*k*_ is the number of individuals in age class *k*. This study predicts the number of individuals in each age class of *R. nymphaeoides* for the next 2, 3, 4, 5, 6, and 7 years, using the number of individuals corresponding to each age class as the base year data.

## 3 Results

### 3.1 Community Characteristics of *R. nymphaeoides*

#### 3.1.1 Composition of Species

The survey results show that the communities of the four *R. nymphaeoides* populations contain a total of 122 vascular plant species, belonging to 55 families and 79 genera (Table 2). Community I comprises 37 species across 25 families and 29 genera, with the most species in the Rosaceae family (5 species), followed by Ericaceae (4 species) and Berberidaceae (3 species), accounting for 13.51%, 10.81%, and 8.11% of the community's total, respectively. Primulaceae, Poaceae, and Symplocaceae each have 2 species, making up 16.22% combined, with the remaining 19 families having one species each. Community II consists of 31 species across 22 families and 28 genera. In this community, the Fagaceae, Rosaceae, and Lauraceae families each have 3 species, representing 29.03% of the community's total; Ericaceae, Betulaceae, and Araliaceae each have 2 species, contributing to 19.35%, with the remaining 16 families having one species each. Community III includes 32 species across 21 families and 29 genera, with the highest number of species again in the Rosaceae family (5 species), followed by Fagaceae (4 species) and Ericaceae (3 species), accounting for 15.63%, 12.50%, and 9.38% of the total, respectively. Fabaceae and Poaceae each have 2 species, accounting for 12.50%, with the remaining 16 families having one species each. Community IV is the most species‐rich, with 60 species across 33 families and 43 genera. The Rosaceae family leads with 7 species, followed by Ericaceae (6 species), Fagaceae (5 species), and Smilacaceae (3 species), representing 11.67%, 10.00%, 8.33%, and 5.00% of the community's total, respectively. Nine families contain 2 species each, and the remaining 20 families each have one species.

**TABLE 2:** Composition of family, genus, and species of vascular plants in *R. nymphaeoides* community.

No.	Family name	Community I		Community II		Community III		Community IV	
		Genus number	Species number	Genus number	Species number	Genus number	Species number	Genus number	Species number
1	Pteridaceae	—	—	1	1	—	—	—	—
2	Thelypteridaceae	—	—	—	—	1	1	—	—
3	Dennstaedtiaceae	1	1	1	1	1	1	—	—
4	Dryopteridaceae	1	1	—	—	—	—	1	1
5	Lauraceae	1	1	3	3	1	1	2	2
6	Styracaceae	1	1	—	—	—	—	—	—
7	Primulaceae	1	2	—	—	—	—	—	—
8	Icacinaceae	—	—	—	—	—	—	1	1
9	Euphorbiaceae	—	—	—	—	—	—	1	1
10	Aquifoliaceae	1	1	—	—	—	—	1	1
11	Fabaceae	1	1	—	—	2	2	—	—
12	Ericaceae	2	4	2	2	2	3	3	6
13	Pittosporaceae	—	—	—	—	—	—	1	1
14	Taxaceae	—	—	1	1	—	—	1	1
15	Juglandaceae	—	—	—	—	1	1	—	—
16	Elaeagnaceae	1	1	—	—	—	—	—	—
17	Daphniphyllaceae	—	—	—	—	—	—	1	1
18	Betulaceae	—	—	1	2	—	—	—	—
19	Hamamelidaceae	—	—	1	1	—	—	—	—
20	Malvaceae	—	—	1	1	—	—	—	—
21	Stachyuraceae	—	—	1	1	—	—	1	1
22	Fagaceae	—	—	2	3	3	4	3	6
23	Orchidaceae	—	—	1	1	—	—	—	—
24	Gleicheniaceae	—	—	—	—	1	1	—	—
25	Loganiaceae	—	—	—	—	—	—	1	1
26	Actinidiaceae	—	—	—	—	—	—	1	1
27	Magnoliaceae	—	—	—	—	—	—	1	1
28	Oleaceae	—	—	—	—	—	—	1	2
29	Vitaceae	1	1	—	—	—	—	—	—
30	Clethraceae	1	1	—	—	—	—	—	—
31	Rubiaceae	1	1	—	—	—	—	—	—
32	Rosaceae	2	5	2	3	4	5	3	7
33	Colchicaceae	1	1	—	—	—	—	1	1
34	Caprifoliaceae	—	—	1	1	1	1	—	—
35	Moraceae	1	1	1	1	1	1	1	2
36	Theaceae	1	1	—	—	1	1	1	1
37	Symplocaceae	1	2	—	—	1	1	1	2
38	Ebenaceae	1	1	—	—	—	—	—	—
39	Iteaceae	—	—	—	—	—	—	1	1
40	Garryaceae	—	—	—	—	—	—	1	1
41	Santalaceae	—	—	—	—	1	1	—	—
42	Asparagaceae	—	—	1	1	—	—	—	—
43	Celastraceae	—	—	1	1	—	—	2	2
44	Sapindaceae	1	1	—	—	—	—	1	2
45	Adoxaceae	—	—	1	1	1	1	1	1
46	Araliaceae	1	1	2	2	1	1	1	1
47	Pentaphylacaceae	1	1	—	—	1	1	2	2
48	Berberidaceae	2	3	1	1	1	1	1	1
49	Hydrangeaceae	1	1	—	—	—	—	1	1
50	Papaveraceae	—	—	—	—	—	—	1	1
51	Rutaceae	1	1	1	1	—	—	—	—
52	Bignoniaceae	—	—	—	—	1	1	—	—
53	Smilacaceae	—	—	—	—	1	1	1	3
54	Cyperaceae	—	—	1	1	—	—	1	2
55	Poaceae	2	2	1	1	2	2	2	2
Total		29	37	28	31	29	32	43	60

#### 3.1.2 Importance Value

The survey results indicate clear dominance of certain species across different strata within the four communities of *R. nymphaeoides* (Table 3). In Community I, the tree layer ranges in height from 2.0 to 8.0 m, with the top three species by importance value being *R. nymphaeoides*, *Clethra delavayi*, and *Lyonia ovalifolia* var. *lanceolata*, accounting for 71.45% of the tree layer importance values. The shrub layer, ranging from 0.2 to 3.0 m in height, has *Fargesia spathacea* , *R. nymphaeoides*, *Skimmia reevesiana*, *Rubus hunanensis*, and *Ardisia crenata* as the top five, making up 72.77% of the shrub layer importance values. The herbaceous layer, with heights from 0.2 to 0.5 m, sees *Oplismenus undulatifolius* , *Monachosorum henryi*, and *Causonis japonica* as the top three, comprising 77.57% of the herbaceous layer importance values.

**TABLE 3:** Top major species in importance value of *R. nymphaeoides* community.

Layer	Community I		Community II		Community III		Community IV	
	Species	Importance value	Species	Importance value	Species	Importance value	Species	Importance value
Tree layer	*Rhododendron nymphaeoides*	46.26	*Quercus phillyraeoides*	34.71	*R. nymphaeoides*	52.63	*R. nymphaeoides*	43.48
	*Clethra delavayi*	14.27	*Carpinus rupestris*	25.75	*L. ovalifolia*	15.17	*Osmanthus fragrans*	8.31
	*Lyonia ovalifolia* var. *lanceolata*	10.92	*Distylium racemosum*	18.09	*Lithocarpus hancei*	12.57	*L. ovalifolia*	7.15
	*Lyonia ovalifolia*	7.74	*R. nymphaeoides*	15.3	*Castanopsis chunii*	7.61	*L. hancei*	6.28
	*Dalbergia hupeana*	6.97	*Carpinus pubescens*	6.14	*Pyrularia edulis*	5.03	*Symplocos ramosissima*	5.64
	*Dendropanax dentiger*	3.28	—	—	*Quercus jenseniana*	4.08	*Sorbus folgneri*	5.31
	*Sorbus megalocarpa*	2.56	—	—	*Eurya nitida*	1.48	*C. chunii*	4.75
	*Rubus malifolius*	2.15	—	—	*Camellia pitardii*	1.43	*Quercus engleriana*	4.38
	*Elaeagnus henryi*	1.4	—	—	—	—	*Lithocarpus megalophyllus*	3.76
	*Rhododendron farrerae*	1.39	—	—	—	—	*Michelia wilsonii* subsp. *szechuanica*	2.44
Shrub layer	*Fargesia spathacea*	44.43	*F. spathacea*	39.61	*Rhododendron simsii*	16.75	*F. spathacea*	25.03
	*R. nymphaeoides*	14.97	*R. nymphaeoides*	11.36	*Stranvaesia davidiana*	9.14	*R. nymphaeoides*	6.17
	*Skimmia reevesiana*	6.19	*Q. phillyraeoides*	3.67	*Rhododendron nymphaeoides*	7.6	*R. simsii*	3.82
	*Rubus hunanensis*	3.59	*Rubus faberi*	3.45	*E. nitida*	6.46	*L. hancei*	3.63
	*Ardisia crenata*	3.59	*C. rupestris*	3.34	*Viburnum fordiae*	5.71	*S. china*	3.63
	*Litsea elongata*	2.02	*Vaccinium dunalianum*	3.34	*Abelia uniflora*	5.33	*Vaccinium japonicum* var. *sinicum*	3.3
	*Symplocos ramosissima*	1.91	*Stachyurus obovatus*	3.22	*L. ovalifolia*	4.56	*L. ovalifolia*	3.25

	*Paederia foetida*	1.91	*Lindera prattii*	3.11	*Smilax china*	4.18	*Machilus chuanchienensis*	3.11
	*Rubus columellaris*	1.91	*Cotoneaster glaucophyllus*	3.11	*S. folgneri*	4.18	*Ternstroemia gymnanthera*	3.06
*Ficus heteromorpha*	1.91	*Quercus gambleana*	1.84	*Symplocos sumuntia*	3.8	*Smilax microphylla*	2.22
Herb layer	*Oplismenus undulatifolius*	40.66	*Carex capilliformis*	48.65	*Miscanthus floridulus*	36.71	*C. capilliformis*	47.28
	*Monachosorum henryi*	23.77	*Epimedium brevicornu*	15.44	*Diplopterygium glaucum*	27.29	*Cyrtomium omeiense*	16.12
	*Causonis japonica*	13.14	*Pteridium revolutum*	15.22	*Parathelypteris glanduligera*	16.18	*Carex cruciata*	12.05
	*Disporum cantoniense*	13.14	*Pteris inaequalis*	12.14	*P. revolutum*	12.8	*Corydalis bungeana*	9.87
	*Dryopteris fuscipes*	9.28	*Ophiopogon japonicus*	4.52	*Incarvillea arguta*	7	*M. floridulus*	7.7
	—	—	*Cymbidium goeringii*	4.05	—	—	*Disporum cantoniense*	6.97

In Community II, the tree layer heights are between 1.0 and 6.0 m, with *Quercus phillyraeoides*, *Carpinus rupestris*, and *Distylium racemosum* leading with 78.55% of the importance values. The shrub layer, ranging from 0.3 to 4.0 m, features *F. spathacea* , *R. nymphaeoides*, *Q. phillyraeoides*, *Rubus faberi*, and *Carpinus rupestris* as the top five, constituting 61.43% of the importance values. The herb layer, at heights of 0.06–0.8 m, ranks *Carex capilliformis*, *Epimedium brevicornu*, and *Pteridium revolutum* as the top three, with 79.31% of the importance values.

Community III features tree layers of 1.0–6.3 m, with *R. nymphaeoides*, *L. ovalifolia*, and *Lithocarpus hancei* as the top three, accounting for 80.37% of tree layer importance values. The shrub layer, ranging from 0.05 to 4 m, sees *Rhododendron simsii*, *Stranvaesia davidiana* , *R. nymphaeoides*, *Eurya nitida* , and *Viburnum fordiae* as the top five, making up 45.66% of the importance values. The herbaceous layer, from 0.06 to 1 m, features *Miscanthus floridulus* , *Diplopterygium glaucum* , and *Parathelypteris glanduligera* as the top three, contributing 80.18% of the importance values.

In Community IV, the tree layer height ranges from 1.0 to 9.2 m, with *R. nymphaeoides*, *Osmanthus fragrans* , and *L. ovalifolia* being the top three, comprising 58.94% of the tree layer importance values. The shrub layer, from 0.03 to 5.5 m, has *F. spathacea* , *R. nymphaeoides*, *R. simsii*, *L. hancei*, and *Smilax china* as the top five, accounting for 42.28% of importance values. The herbaceous layer, from 0.03 to 0.5 m, features *C. capilliformis*, *Cyrtomium omeiense*, and *Carex cruciata* as the top three, making up 75.45% of the importance values.

All four communities share seven families, four genera, and two species, with Jaccard similarity indexes of 0.0745, 0.0320, and 0.0127, respectively, all below 0.25. This indicates very low similarity among the four communities of *R. nymphaeoides*, with significant differences in the species, genera, and families of the plants within each community.

#### 3.1.3 Diversity Analysis

By calculating the species diversity indices at different levels (Table 4), it is observed that in all four communities, the Margalef richness index shows a pattern of shrub layer > tree layer > herb layer. This is mainly because the shrub layer not only includes saplings and young trees from the tree layer but also has unique species of its own, making it more diverse. In Community I, the Simpson dominance index and Shannon‐Wiener diversity index are higher in the tree layer than in the shrub and herb layers, while the Pielou evenness index shows the herb layer > tree layer > shrub layer, indicating a more even distribution of species in the herb layer of Community I. In Community II, the Simpson dominance index and Pielou evenness index are highest in the tree layer, followed by the herb and shrub layers. The low evenness index in the shrub layer of Community II suggests possible species competition or environmental adaptability issues. In Communities III and IV, the shrub layer's Simpson dominance index, Shannon‐Wiener diversity index, and Pielou evenness index are higher than those of the tree and herb layers, indicating greater differences in species abundance in the shrub layers of these communities. Overall, Community IV has the highest Margalef richness index and Shannon‐Wiener diversity index, while Community III has the highest Simpson dominance index and Pielou evenness index, suggesting that Community IV has the highest species diversity and Community III has the best evenness in species distribution, with Community II having the lowest diversity. Moreover, a Kruskal–Wallis test revealed that the species diversity among the four communities does not show statistical significance (*p* > 0.05).

**TABLE 4:** Diversity index of different layers of *R. nymphaeoides* community.

Community	Layer	*R*	*D*	*H*′	*J*_*e*_
I	Tree layer	2.7107	0.7481	1.8373	0.6962
	Shrub layer	3.1286	0.3609	0.9732	0.3249
	Herb layer	0.9294	0.6535	1.2865	0.7994
	Community	5.5865	0.6720	1.8995	0.5260
II	Tree layer	1.5157	0.8242	1.5125	0.9397
	Shrub layer	3.9313	0.4436	1.1943	0.3710
	Herb layer	0.9343	0.4932	0.9931	0.5543
	Community	4.6071	0.7030	1.8094	0.5269
III	Tree layer	1.6961	0.6467	1.4003	0.6734
	Shrub layer	4.7178	0.9122	2.7338	0.8602
	Herb layer	0.9447	0.7050	1.3358	0.8300
	Community	5.5672	0.9273	2.9217	0.8430
IV	Tree layer	3.1949	0.7671	2.0090	0.7419
	Shrub layer	8.6084	0.7803	2.5607	0.6580
	Herb layer	1.1691	0.5325	1.1027	0.6155
	Community	9.7833	0.8820	2.9540	0.7215

Abbreviations: *D*, Simpson dominance index; *H'*, Shannon‐Wiener diversity index; *J*, Pielou evenness index; *R*, Margalef richness index.

### 3.2 Population Characteristics of *R. nymphaeoides*

#### 3.2.1 Population Age Structure for *R. nymphaeoides*

**FIGURE 3:**
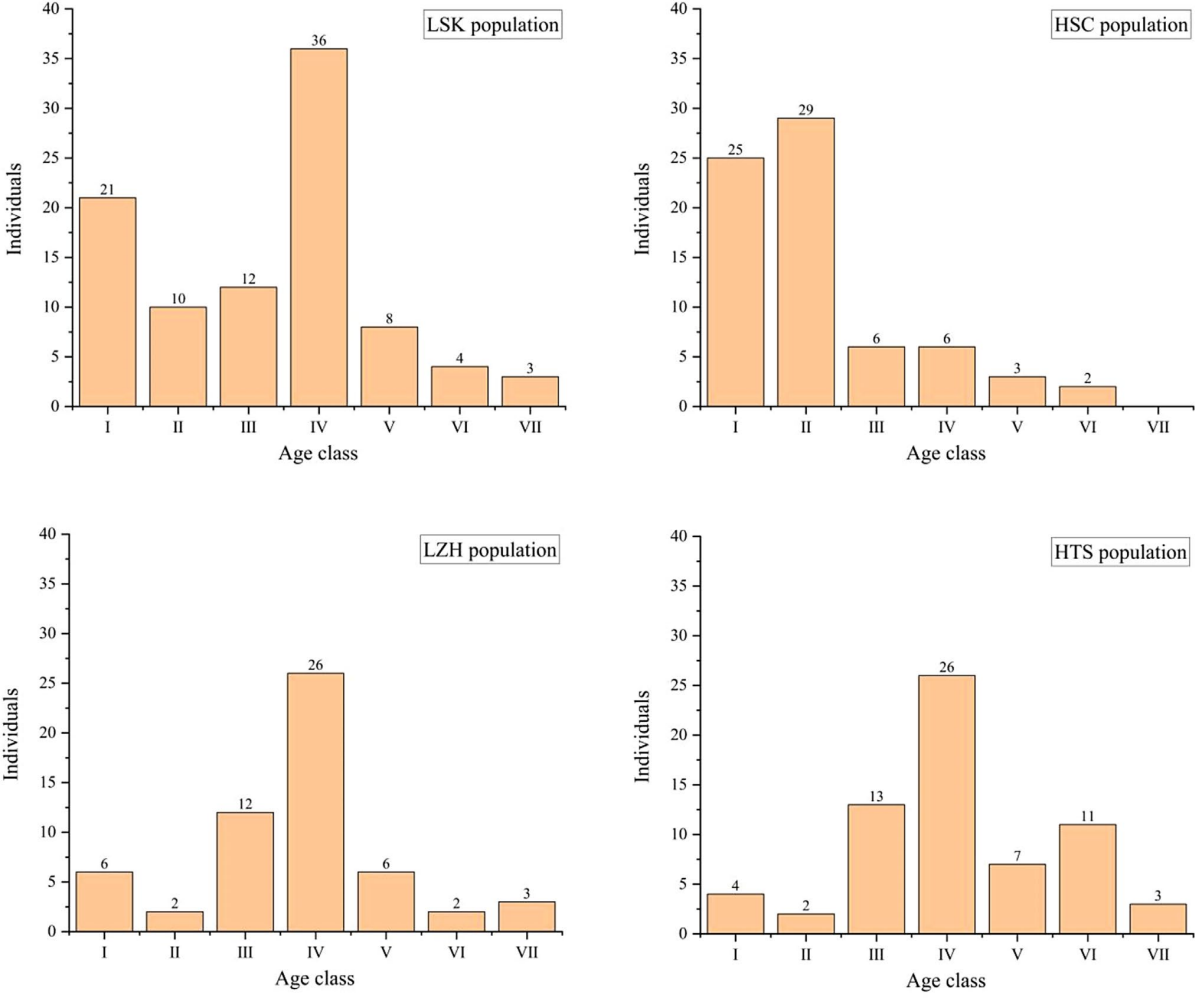
Age structure of *R. nymphaeoides* populations.

During surveys at four distribution sites, we identified a total of 288 individuals of *R. nymphaeoides*, with 94 in the LSK population, 71 in the HSC population, 57 in the LZH population, and 66 in the HTS population. The age structure of *R. nymphaeoides* populations reveals (Figure 3) that in the LSK population, age classes I to II represent 32.98%, III to IV 51.06%, V to VI 12.76%, and VII 3.19%. The mid‐age classes have the largest proportion, followed by the younger classes, with the diameter structure resembling a “spindle shape,” indicating a stable population. In the HSC population, age class I accounts for 35.21%, II 40.85%, III to IV 16.90%, V to VI 7.04%, with no individuals reaching age class VII. This population has the highest proportion in the younger and mid‐age classes, and its diameter structure exhibits an “inverted J shape,” indicating a growing population. The LZH population shows that age classes I to II comprise 14.04%, III to IV 66.67%, V to VI 14.04%, and VII 5.26%, with the middle age classes having the highest proportion, and the diameter structure resembling a “spindle shape,” indicating a stable population. Finally, the HTS population has age classes I to II at 9.09%, III to IV at 59.09%, V to VI at 27.27%, and VII at 4.55%. Here, the mid‐age classes dominate, followed closely by the mature classes, with a deficiency in younger individuals. This population's diameter structure also takes a “spindle shape,” indicating a stable population, but there is a risk of decline.

#### 3.2.2 Spatial Distribution Patterns for *R. nymphaeoides*

As shown in Table 5, the spatial structure of *R. nymphaeoides* populations at each distribution point exhibits a clustered distribution. The LSK population has the highest values of *C*, *I*, M*, and *P*, while the HSC population has the highest *K* value. Considering all indices, the degree of aggregation for the four populations is ranked as follows: LSK population > HTS population > LZH population > HSC population.

**TABLE 5:** Spatial patterns of *R. nymphaeoides* population distributed in different locations.

Population	Mean	Variance	*C*	*K*	*I*	M*	*P*	Distribution pattern
LSK	5.875	11.183	1.904	6.502	2.294	6.779	1.154	Clustered distribution
HSC	4.438	6.929	1.562	7.903	2.016	4.999	1.127	Clustered distribution
LZH	3.563	5.196	1.458	7.770	2.028	4.021	1.129	Clustered distribution
HTS	4.125	6.383	1.547	7.535	2.043	4.672	1.133	Clustered distribution

Abbreviations: *C*, diffusion index; *I*, clumping index; *K*, negative binomial index; M*, average congestion; *P*, agglomeration index.

#### 3.2.3 Population Static Life Table

**FIGURE 4:**
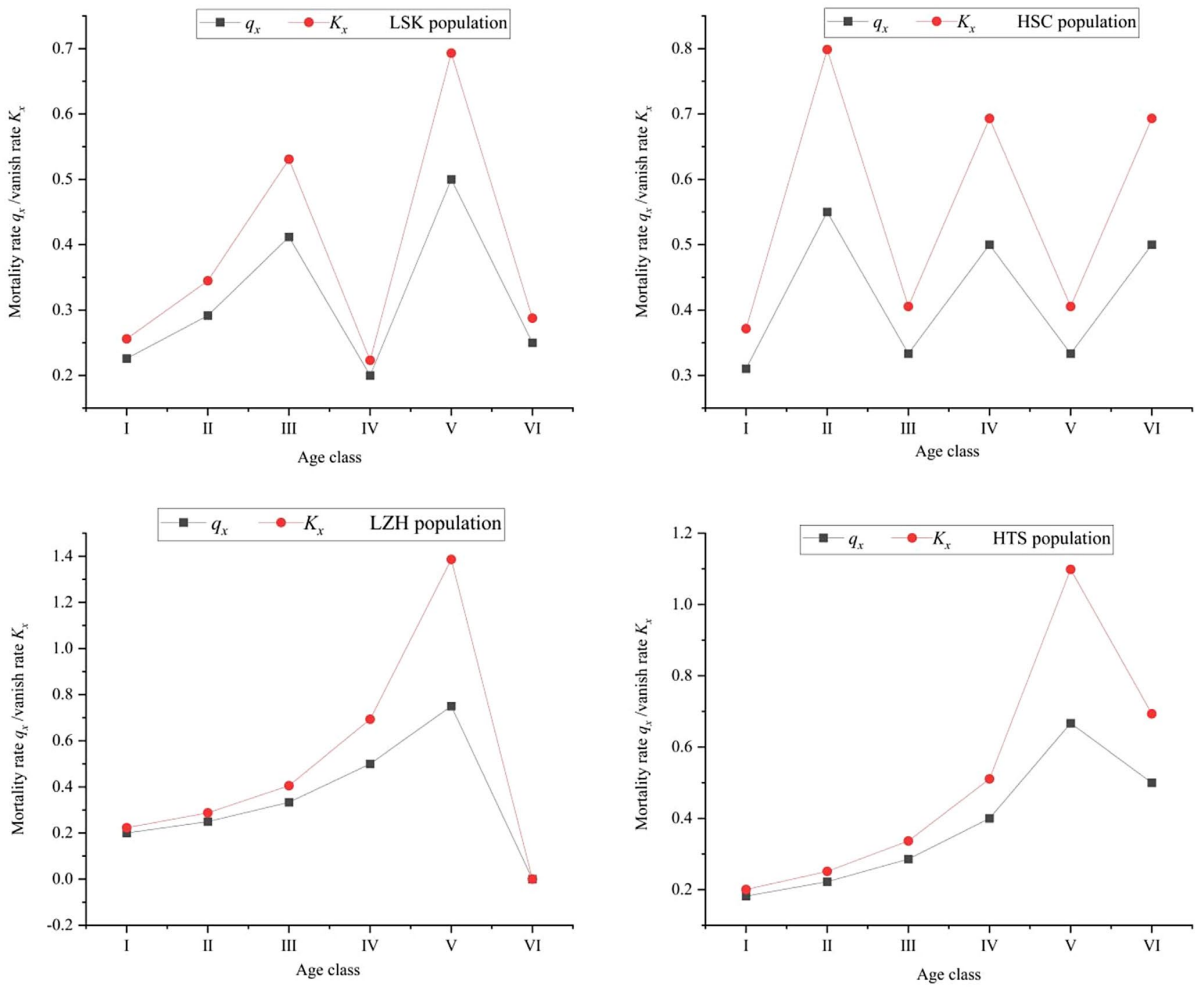
Mortality and vanish rate curves of *R. nymphaeoides* populations.

According to the static life table of *R. nymphaeoides* population (Table 6), as age classes increase, the number of surviving individuals of *R. nymphaeoides* tends to decrease. The youngest individuals (age classes I and II) exhibit the highest life expectancy(*e*_*x*_), which gradually decreases with age. However, the LSK and HSC populations show fluctuations in life expectancy(*e*_*x*_) between age classes II and IV. The mortality and extinction rate curves (Figure 4) indicate that the mortality and extinction rates for the LSK and HSC populations follow a pattern of increase–decrease–increase–decrease with increasing age classes. There is a higher mortality rate during the transition from young to middle age classes, indicating significant losses during this stage for these two populations. In contrast, the LZH and HTS populations show an increase in mortality and extinction rates with increasing age classes, with a sharp rise between age classes IV and V, reaching a peak at age class V, suggesting that the living environment has a significant impact on individuals during this stage.

**TABLE 6:** The static life table of *R. nymphaeoides* populations.

Population	Age class	*A*_*x*_	*a*_*x*_	*l*_*x*_	ln*l*_*x*_	*d*_*x*_	*q*_*x*_	*L*_*x*_	*T*_*x*_	*e*_*x*_	*K*_*x*_	*S*_*x*_
LSK	I	21	31	1000	6.908	226	0.226	887.097	2629	**2.629**	0.256	0.774
	II	10	24	774	6.652	226	0.292	661.290	1742	2.250	0.345	0.708
	III	12	17	548	6.307	226	0.412	435.484	1081	1.971	0.531	0.588
	IV	36	10	323	5.776	65	0.200	290.323	645	2.000	0.223	**0.800**
	V	8	8	258	5.553	129	0.500	193.548	355	1.375	**0.693**	0.500
	VI	4	4	129	4.860	32	0.250	112.903	161	1.250	0.288	0.750
	VII	3	3	97	4.572			48.387	48	0.500		
HSC	I	25	29	1000	6.908	310	0.310	844.828	1914	**1.914**	0.372	**0.690**
	II	29	20	690	6.536	379	0.550	500.000	1069	1.550	**0.799**	0.450
	III	6	9	310	5.738	103	0.333	258.621	569	1.833	0.405	0.667
	IV	6	6	207	5.332	103	0.500	155.172	310	1.500	0.693	0.500
	V	3	3	103	4.639	34	0.333	86.207	155	1.500	0.405	0.667
	VI	2	2	69	4.234	34	0.500	51.724	69	1.000	0.693	0.500
	VII		1	34	3.540			17.241	17	0.500		
LZH	I	6	20	1000	6.908	200	0.200	900.000	2600	**2.600**	0.223	0.800
	II	2	16	800	6.685	200	0.250	700.000	1700	2.125	0.288	0.750
	III	12	12	600	6.397	200	0.333	500.000	1000	1.667	0.405	0.667
	IV	26	8	400	5.991	200	0.500	300.000	500	1.250	0.693	0.500
	V	6	4	200	5.298	150	0.750	125.000	200	1.000	**1.386**	0.250
	VI	2	1	50	3.912	0	0.000	50.000	75	1.500	0.000	**1.000**
	VII	3	1	50	3.912			25.000	25	0.500		
HTS	I	4	22	1000	6.908	182	0.182	909.091	2818	**2.818**	0.201	**0.818**
	II	2	18	818	6.707	182	0.222	727.273	1909	2.333	0.251	0.778
	III	13	14	636	6.456	182	0.286	545.455	1182	1.857	0.336	0.714
	IV	26	10	455	6.119	182	0.400	363.636	636	1.400	0.511	0.600
	V	7	6	273	5.608	182	0.667	181.818	273	1.000	**1.099**	0.333
	VI	11	2	91	4.510	45	0.500	68.182	91	1.000	0.693	0.500
	VII	3	1	45	3.817			22.727	23	0.500		

*Note:* The bold values are the maximum values for each population.

Abbreviations: *A*_*x*_, actual number of surviving individuals; *a*_*x*_, the correction value of *A*_*x*_; *d*_*x*_, standardized deaths from *x* to *x +* 1 age class; *e*_*x*_, average life expectancy; *K*_*x*_, vanish rate; ln*l*_*x*_, logarithm of *l*_*x*_; *l*_*x*_, standardized number of the number of surviving individuals in *x* age class; *L*_*x*_, survived individuals from *x* to *x* + 1 age class; *q*_*x*_, mortality rate; *S*_*x*_, survival rate; *T*_*x*_, total number of surviving individuals greater than or equal to the *x* age class. The same below.

#### 3.2.4 Survival Curves for *R. nymphaeoides*

**FIGURE 5:**
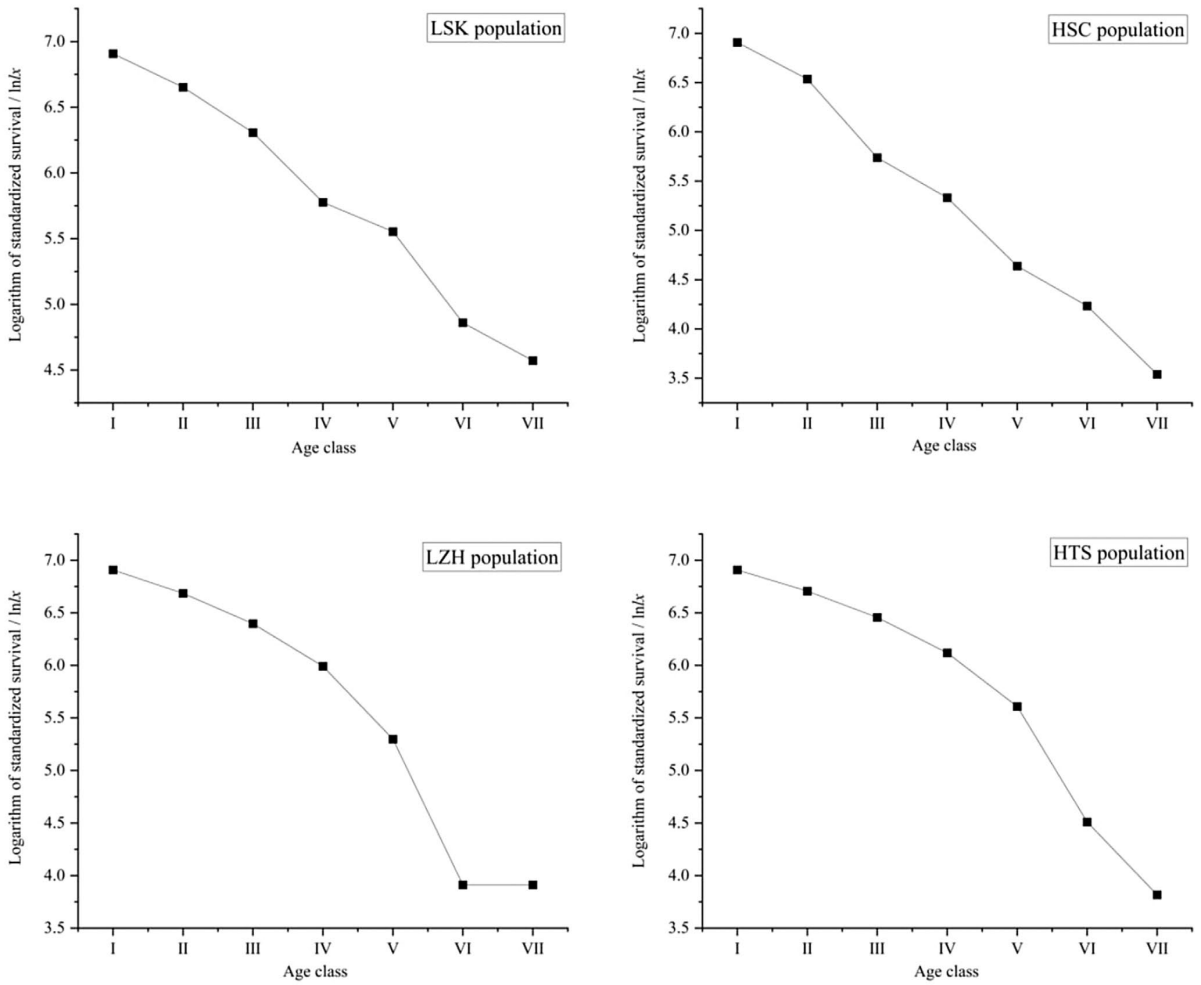
Survival curve of *R. nymphaeoides* populations.

Using the logarithm of standardized surviving individuals as the vertical axis, the survival curve of the *R. nymphaeoides* population was plotted (Figure 5). The results show that the survival curve of the *R. nymphaeoides* population trends downward with increasing age of the population. However, the LZH and HTS populations have a relatively gradual decline in the early stages, with the rate of decline increasing beginning from age class V. The survival curves of the *R. nymphaeoides* population were analyzed using exponential and power function models, revealing that both models produced very significant results (Table 7). However, the exponential model's *R* ^2^ and *F* values were higher than those of the power function model, indicating that the exponential function provided a better fit. The results indicate that the population survival curve conforms to the Deevey‐II type. The model validation results are consistent with the trends observed in the survival curve.

**TABLE 7:** Test models of survival curves of *R. nymphaeoides* populations.

Population	Survival curve equation	*R* ^2^	*F*	Type of survival curve
LSK	*y* = 7.6376e^−0.071x^	0.975	196.302	Deevey‐II
	*y* = 7.4075x^−0.209^	0.842	26.694	
HSC	*y* = 8.0036e^−0.11x^	0.984	309.903	Deevey‐II
	*y* = 7.6554x^−0.326^	0.862	31.349	
LZH	*y* = 8.3312e^−0.106x^	0.890	40.257	Deevey‐II
	*y* = 7.851x^−0.299^	0.713	12.421	
HTS	*y* = 8.2794e^−0.097x^	0.8817	37.265	Deevey‐II
	*y* = 7.7899x^−0.268^	0.679	10.577	

#### 3.2.5 Population Viability for *R. nymphaeoides*

**FIGURE 6:**
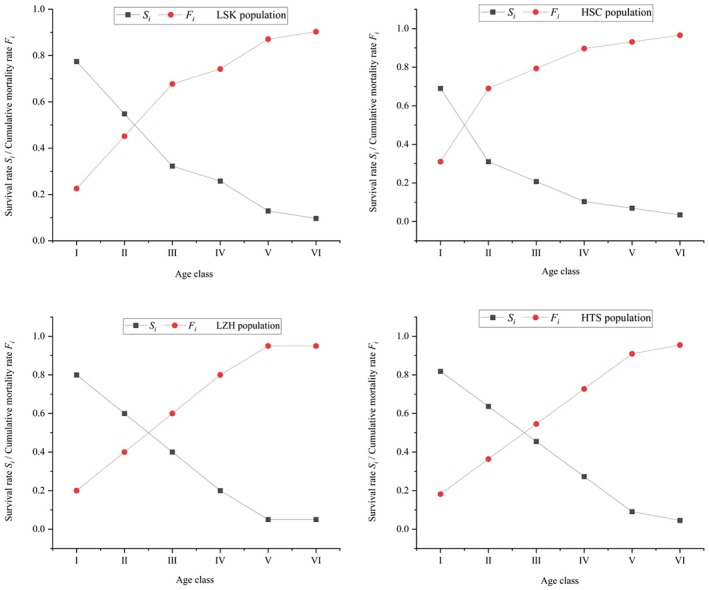
Survival rate and cumulative mortality rate of *R. nymphaeoides* populations.

According to the survival and cumulative mortality curves of the *R. nymphaeoides* population (Figure 6), as the age classes increase, the survival rate of the population shows a decreasing trend, while the cumulative mortality rate increases. For all four populations, the cumulative mortality rates complement their survival rates. The HSC population reaches equilibrium between the survival and cumulative mortality rates in age classes I to II, indicating poor survival ability in seedlings and low survival rates, leading to an earlier onset of the population's decline phase. The LSK, LZH, and HTS populations reach this equilibrium between age classes II and III, entering the decline phase relatively later.

**FIGURE 7:**
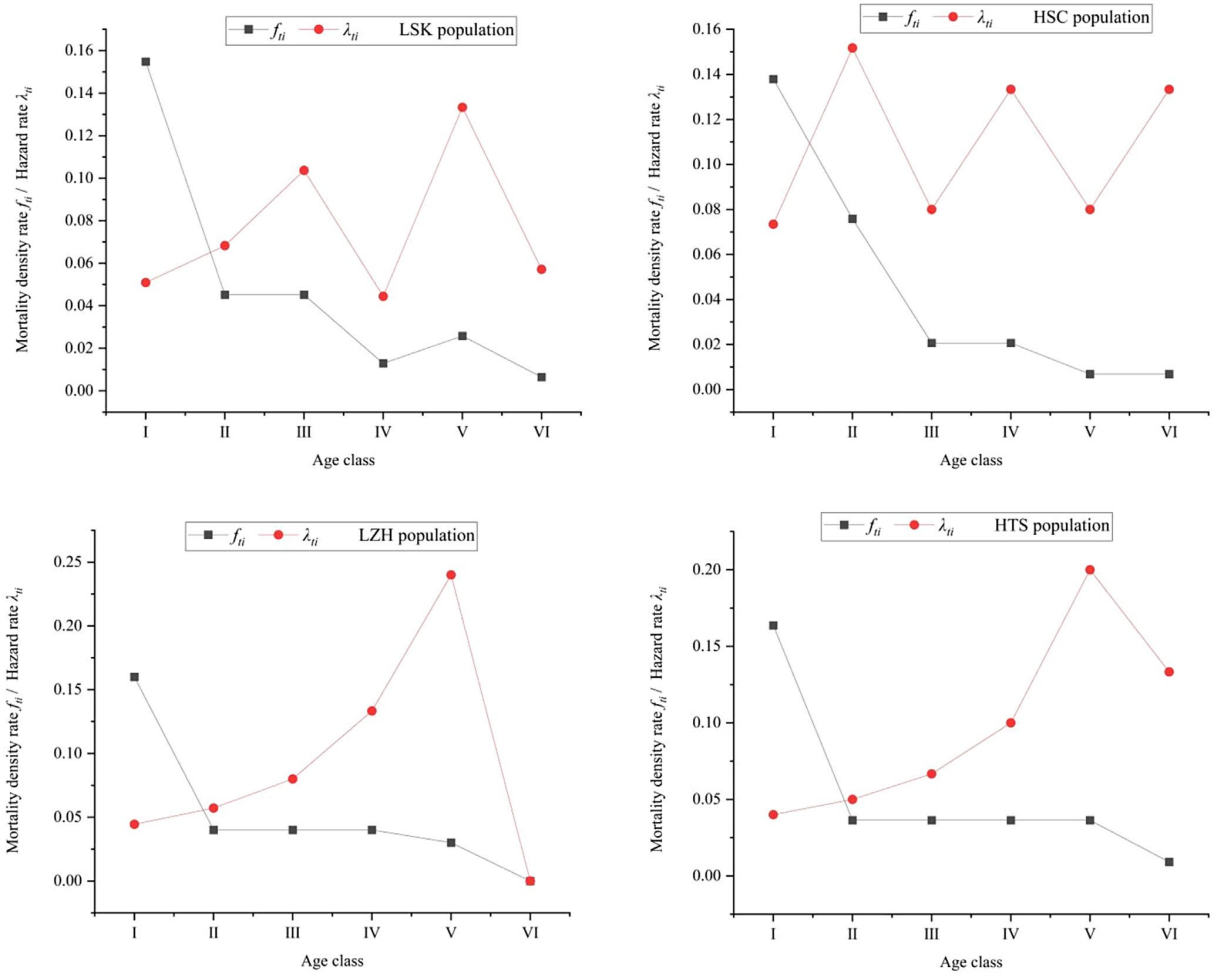
Mortality density and hazard rates of *R. nymphaeoides* populations.

According to the mortality density and hazard rate curves of *R. nymphaeoides* population (Figure 7), the mortality density function decreases sharply in the juvenile phase (age classes I to II) and then stabilizes, while the hazard rate increases with age. The hazard rate reaches its maximum in age class II for the HSC population, indicating significant survival pressures in the early stages for this group. For the LSK, LZH, and HTS populations, the hazard rate peaks in age class V, indicating that these populations face significant survival pressures after reaching age class V.

#### 3.2.6 Population Dynamics Quantification for *R. nymphaeoides*

As shown in Table 8, the *V*_2_ and *V*_3_ values for the LSK population are negative, indicating a declining structural dynamic between age classes II to III and III to IV. The HSC population only has a negative *V*_1_ value, indicating a declining dynamic between age classes I and II. Both the LZH and HTS populations exhibit negative values in earlier and later stages, showing significant fluctuations in population structure dynamics. The quantity dynamic change indices *V*_*pi*_ and $V^{\prime }\mathrm{pi}$ for all four populations are greater than 0, suggesting that the four *R. nymphaeoides* populations mostly exhibit growth dynamics over time; even with external disturbances, the populations manage to maintain a state of growth. The LSK population shows the most optimal dynamics, while the HTS population shows relatively the worst dynamics. The probability of random disturbance risk *P*_max_ ranges between 4.76 and 8.33, indicating that all four populations have poor stability and are highly sensitive to external disturbances.

**TABLE 8:** Dynamic index of the age structure of *R. nymphaeoides* populations.

Dynamic index class	Dynamic index value (%)			
	LSK population	HSC population	LZH population	HTS population
*V*_1_	52.38	−13.79	66.67	50
*V*_2_	−16.67	79.31	−83.33	−84.62
*V*_3_	−66.67	0	−53.85	−50
*V*_4_	77.78	50	76.92	73.08
*V*_5_	50	33.33	66.67	−36.36
*V*_6_	25	—	−33.33	72.73
*V*_*pi*_	37.73	34.13	35.57	28.99
$V_{\mathrm{pi}}^{\prime }$	1.8	2.84	2.54	2.07
*P*_max_	4.76	8.33	7.14	7.14

Abbreviations: $V_{\mathrm{pi}}^{\prime }$, Dynamic index with external interference; *P*_max_, The maximum probability in random disturbance; *V*_1_–*V*_6_, Dynamic index of quantitative change between adjacent age classes; *V*_*pi*_, Dynamic index without external interference.

#### 3.2.7 Time Series Modeling and Forecasting

The results of the time series forecast for population dynamics (Table 9) show that after 2–7 age classes, the HSC population exhibits a clear increasing trend across all age classes. In contrast, the LSK, LZH, and HTS populations show an increase in numbers in age classes I–II, a decrease in age classes III–IV, and an overall growth trend in age classes V–VII, with a slight decline in the later stages. This indicates that in the coming period, under stable environmental conditions, the HSC population is expected to maintain a growth trend. However, the LSK, LZH, and HTS populations, due to a lack of sufficient recruitment of juveniles, will see the existing individuals gradually transition to maturity. This will result in a scarcity of juvenile individuals and an increase in mature individuals, leading the populations to exhibit a declining trend.

**TABLE 9:** Time sequence prediction of number dynamics of *R. nymphaeoides* populations.

Population	Age class	*A*_*x*_	$M_{2}^{\left[1\right]}$	$M_{3}^{\left[1\right]}$	$M_{4}^{\left[1\right]}$	$M_{5}^{\left[1\right]}$	$M_{6}^{\left[1\right]}$	$M_{7}^{\left[1\right]}$
LSK	I	21						
	II	10	16					
	III	12	11	14				
	IV	36	24	19	20			
	V	8	22	19	17	17		
	VI	4	6	16	15	14	15	
	VII	3	4	5	13	13	12	13
HSC	I	25						
	II	29	27					
	III	6	18	20				
	IV	6	6	14	17			
	V	3	5	5	11	14		
	VI	2	3	4	4	9	12	
	VII		1	2	3	3	8	10
LZH	I	6						
	II	2	4					
	III	12	7	7				
	IV	26	19	13	12			
	V	6	16	15	12	10		
	VI	2	4	11	12	10	9	
	VII	3	3	4	9	10	9	8
HTS	I	4						
	II	2	3					
	III	13	8	6				
	IV	26	20	14	11			
	V	7	17	15	12	10		
	VI	11	9	15	14	12	11	
	VII	3	7	7	12	12	10	9

Abbreviations: *A*_*x*_, actual number of surviving individuals; $M_{x}^{\left[1\right]}$ represents the predicted value of the population at age class *x* after it has experienced the next age classes of time.

## 4 Discussion

### 4.1 Status of Community of *R. nymphaeoides*

Species composition is one of the most crucial characteristics of a community, forming the basis of community formation and serving as a key indicator to distinguish different communities. The species composition and diversity of a community are primarily influenced by environmental factors, human activities, and the developmental stage of the community (Zhan et al. 2023). Existing research has shown that the genetic distance among populations of *R. nymphaeoides* is significantly positively correlated with geographical distance (*r* = 0.8456, *p* = 0.0021), conforming to the isolation‐by‐distance model (Luo et al. 2025). This indicates that the four communities of *R. nymphaeoides* are located in the three major mountain ranges of Miaoling, Wumengshan, and Daloushan, and their distribution is fragmented due to geographical barriers. Community IV has 60 plant species, the highest Margalef richness index, and Shannon‐Wiener diversity index, indicating the greatest species diversity. Community I has 37 plant species, with diversity indices at a moderate level. Community III has 32 plant species, the highest Simpson dominance index, and Pielou evenness index, suggesting the best evenness in species distribution. Community II has only 31 species, the lowest Margalef richness index, and Shannon‐Wiener diversity index, indicating the lowest species diversity. This variation is partly because the four communities are affected differently by human disturbances; Community IV is located at the Hu Tou Mountain viewing platform in Gulin County, Sichuan Province, and Community I is in the Luosike Scenic Area in Duyun City, Guizhou Province, both experiencing frequent human disturbances and higher spatial heterogeneity, which can accommodate more coexisting plants, aligning with the intermediate disturbance theory (Yao et al. 2017). On the other hand, the four communities are located in different habitats; Community II is situated on a mountain slope with large areas of exposed rock, leading to low plant richness. In the four communities, the dominance of the arboreal, shrub, and herbaceous layers is evident, with tropical elements predominantly influencing the geographical composition of the families (e.g., Rosaceae, Ericaceae, Fagaceae) and genera. However, the families, genera, and species within the communities significantly differ, complying with the richness patterns typical of subtropical high mountain vegetation. This is partly because all four communities are located in the southwestern part of China, a region with low latitude and one of the richest in solar and thermal resources, dominated by tropical components, fitting its mid‐subtropical monsoon climate and geographical conditions (Zhang et al. 2018). Additionally, the differences in species types and quantities within the communities are caused by varying degrees of human disturbance, different successional stages, and small environmental variations (Hao et al. 2021). From the importance values of different layers, *R. nymphaeoides* plays a dominant role in the tree and shrub layers of all four communities, significantly influencing the structure and formation of the environment. Although the abundance of *R. nymphaeoides* within the community is high, the individuals are relatively small in height and DBH, placing them at a disadvantage in competition for resources and making them prone to being outcompeted. Should the *R. nymphaeoides* population be damaged, it would inevitably lead to changes in community properties and environmental conditions. Thus, *R. nymphaeoides* plays a critical role in the stability of its ecosystem.

### 4.2 Status of Populations of *R. nymphaeoides*

Population structure is one manifestation of a plant's adaptability to environmental conditions, reflecting the growth and development processes within the population. The dynamic changes in population numbers can reveal the composition of individuals at different stages, shedding light on the population's current survival status and regeneration strategies (Xu et al. 2021; Gao et al. 2017). This study shows that the LSK population has a high proportion of young individuals and the highest proportion of middle‐aged individuals, characterizing it as a stable population. In contrast, the HSC population has the largest proportion of young individuals and fewer older individuals, with an age structure resembling an inverted J‐shape, indicative of a growing population, with notably high mortality rates during the transition from young to middle age. The LZH and HTS populations have few young individuals and a high proportion of middle‐aged individuals, with a spindle‐shaped age structure, facing significant risks of decline. All four populations exhibit a Deevey‐II type survival curve, characterized by high seedling mortality rates, consistent with studies on *Rhododendron rex* (Yang et al. 2020), *Rhododendron feddei* (Jiang et al. 2020), and *Rhododendron pudingenese* (Zhao et al. 2024). The four populations have high life expectancy at age classes I and II, but actual survival numbers are low, suggesting that *R. nymphaeoides* populations undergo strong environmental selection at these stages, with those that survive possessing enhanced survival capabilities, a characteristic similar to findings in *R. aureum* (Jin et al. 2017). All four populations show a clumped spatial distribution, with the degree of clustering in the order of LSK, HTS, LZH, and HSC populations, corresponding to their age structures, indicating that the structure of *R. nymphaeoides* populations is constrained by habitat and biological characteristics. On the one hand, the extremely small, chaff‐like seeds of *R. nymphaeoides* and its typical heterophyllous structure result in seeds dispersing over short distances under the influence of wind and gravity, leading to clumped distributions and intense intraspecific competition, adversity adaptation, and environmental selection for seedlings, as limited space and resources are major constraints on population regeneration (Xu et al. 2016). On the other hand, as a shrub or small tree, *R. nymphaeoides* is at a competitive disadvantage for light within the community, and the thick layer of fallen branches and leaves in the relatively closed understory often fails to meet the light conditions required for seed germination (Yang et al. 2019). Therefore, it is inferred that the low natural germination rate of *R. nymphaeoides* seeds, weak competitiveness of seedlings, and site conditions are the main reasons for the low number of juvenile individuals and the difficulty in population renewal and development.

### 4.3 Population Dynamics and Trends of *R. nymphaeoides*

Survival function analysis, population dynamic change indices, and time‐series forecasting can further reflect the patterns and distribution of population numbers across time and space, revealing the interactions between species and their living environments (Xu et al. 2016). The survival function indicates that the HSC population enters a declining phase early in its juvenile stage, with the hazard rate peaking at age class II. This aligns with its characteristics of high mortality in the juvenile stage and fewer individuals in the middle and mature stages, consistent with findings in studies of *Hopea chinensis* population, possibly due to the poor resistance of *R. nymphaeoides* juveniles to external environmental disturbances, leading to significant mortality during the transformation from seedlings to saplings; LSK, LZH, and HTS populations reach equilibrium between survival and cumulative mortality rates between age classes II and III, entering the declining phase relatively later, with the hazard rate peaking at age class V. This suggests that these three populations face significant survival pressures in the mature stage, consistent with research on the *Syringa pinnatifolia* population (Jiang et al. 2018), possibly due to reaching a physiological age of death or due to environmental impacts such as specific ecological niche characteristics which make it difficult for them to transition from middle to mature age classes. The dynamic change indices *V*_*pi*_ and $V^{\prime }\mathrm{pi}$ for all four populations are greater than 0, with $V^{\prime }\mathrm{pi}$ significantly lower than *V*_*pi*_, and the probability of maximum risk of random disturbance, *P*_max_ is between 4.76 and 8.33, indicating that *R. nymphaeoides* populations generally belong to a low disturbance‐resistant growth population and are highly sensitive to disturbances. Notably, the dynamic change indices for age classes I–III are overall negative, indicating a clear decline in the juvenile phase. Time‐series forecasting shows that after 2–7 age classes, the HSC population shows a clear increasing trend in numbers at all age classes, similar to findings with *C. granthamiana* (Lin et al. 2023), suggesting that given stable environmental conditions, the numbers of the HSC population can remain relatively stable and even increase; meanwhile, LSK, LZH, and HTS populations show an increase in numbers from age classes I to II, a decrease from III to IV, and a generally increasing trend from V to VII, which corresponds with the negative values observed in the dynamic indices for age classes V to VII. This suggests that these three *R. nymphaeoides* populations are moving toward aging, and if juvenile recruitment does not occur timely, the populations are at risk of decline, similar to conclusions drawn from research by Zhu et al. (2024) on *R. griersonianum*.

### 4.4 Conservation Strategies for *R. nymphaeoides*

The conservation of endangered plants involves not only adequately protecting already diminished populations but also alleviating or reversing the critical factors that threaten the continuation of these species (Huang 2020). The four populations of *R. nymphaeoides* share similarities and differences in their structure and dynamics. A common feature is that the highest mortality rates occur when transitioning from young and middle‐aged stages to mature stages, and all are highly sensitive to external disturbances. The differences lie in the pressures faced by the young age classes: the HSC population has a relatively larger number of young individuals but a higher mortality rate, leading to a smaller proportion transitioning to middle age; the LSK, LZH, and HTS populations are notably lacking in young individuals, making population renewal difficult and tending toward aging. Field surveys have found that the HSC population is the smallest and is located on steep cliffs at an altitude of nearly 1700 m. The absence of birds and animals that could facilitate seed dispersal makes it difficult for them to engage in genetic exchange with external populations, placing significant selective pressure on the HSC population. Additionally, the high canopy closure within the community, with large‐diameter tree species such as *Quercus phillyraeoides* and *Carpinus pubescens*, occupies the living space of *R. nymphaeoides* and further restricts its growth. For the LSK and HTS populations located within scenic areas, tourism and frequent human disturbances have harmed their habitats and greatly affected the regeneration of *R. nymphaeoides* saplings. Therefore, the conservation of *R. nymphaeoides* should adopt different protection strategies based on actual conditions. In light of this, the following hypothetical recommendations, based on the population structure, ecological characteristics, and conservation status of *R. nymphaeoides*, are proposed for consideration: First, it is recommended that each of the four populations correspond to distinct conservation management units. Since the HTS and LZH populations are already located within the Huangjing Lao Forest Nature Reserve, conservation management units for the LSK and HSC populations should be established as soon as possible to strengthen the protection of this endangered species. Second, integrate ex situ conservation with reintroduction strategies. By assessing the ecological factors of the current habitat of *R. nymphaeoides*, suitable relict areas should be located for near‐site ex situ conservation of seedlings to expand the population distribution of *R. nymphaeoides*. Additionally, further research should be conducted on the asexual reproduction techniques of *R. nymphaeoides*, such as tissue culture and cutting, to achieve rapid propagation. After the seeds collected from the field are cultivated in relocated sites, the seedlings should be reintroduced to the original locations to achieve population restoration and the natural reintroduction of artificially cultivated populations, ultimately preventing the extinction of this species. Third, specific measures such as thinning should be adopted for the HSC population to reduce canopy closure and provide adequate light for saplings, thereby improving their survival rates.

## 5 Conclusion

In this study, we assessed the current status of the *R. nymphaeoides* populations and the communities they inhabit. The results indicate that: (1) the four *R. nymphaeoides* communities are comprised of 122 species of vascular plants, belonging to 55 families and 79 genera. The arboreal, shrub, and herbaceous layers show clear dominance, and the geographic components of the families and genera are predominantly tropical, which significantly influences the structure of the communities and the formation of their environments. (2) The four *R. nymphaeoides* populations exhibit spatially clustered distributions. The survivorship curves are all of the Deevey‐II type, indicating high mortality rates among seedlings. Populations at LSK, LZH, and HTS show a clear lack of young individuals, making population renewal challenging and leading to an aging population trend. The HSC population has a relatively higher number of young individuals, but also greater mortality rates, with ongoing constraints in the transition from seedlings to saplings. (3) Factors such as the extremely low natural germination rates of seeds, weak competitiveness of seedlings, exclusive growth in limestone mountains above 1500 m with thin soil layers, and frequent human disturbances are primarily responsible for the challenges in population renewal and development. (4) Strategies that combine in situ conservation, ex situ conservation, and reintroduction should be implemented to achieve population restoration and the natural reintroduction of artificially cultivated populations, ultimately preventing the extinction of this species.

## Author Contributions

**Jun Luo:** conceptualization (equal), data curation (equal), investigation (equal), methodology (equal), visualization (equal), writing – original draft (equal), writing – review and editing (equal). **Xiaoyong Dai:** formal analysis (equal), investigation (equal), resources (equal), writing – review and editing (equal). **Jin Chen:** validation (equal), visualization (equal). **Congjun Yuan:** supervision (equal), writing – review and editing (equal). **Haodong Wang:** validation (equal), visualization (equal). **Dali Luo:** investigation (equal), visualization (equal).

## Conflicts of Interest

The authors declare no conflicts of interest.

## Supporting information


**Table S1.** Community survey data of *Rhododendron nymphaeoides*.

**Table S2.** Population survey data of *Rhododendron nymphaeoides*.

## Data Availability

The data that supports the findings of this study are available in the [Link], [Link] of this article.
